# Implementing blended learning for clinician teachers: a qualitative study identifying their needs and the impact on faculty development initiatives

**DOI:** 10.12688/mep.19243.1

**Published:** 2022-08-16

**Authors:** Elizabeth Kanita Brits, Elize Archer, Sonja Strydom

**Affiliations:** 1 Faculty of Medicine and Health Sciences, Centre for Health Professions Education, Stellenbosch University, Francie van Zijl Drive, Parow, Cape Town, 7505, South Africa

**Keywords:** learning technologies, blended learning, faculty development, digital literacy, 21st century learning

## Abstract

Background: Learning Technologies has been a fast-growing field in Health Professions Education (HPE). Approaches to teaching, learning and assessment have been increasingly influenced by learning technologies which requires HPE teachers to adapt their teaching practices and, with that, identify areas for professional development.

The implementation of blended learning in HPE, has shown improvements in student performance. However, it seems as if there are challenges with the implementation of a blended learning approach and that there might be some needs that clinical teachers have that are not being addressed in order to implement blended learning successfully.

Methods: We used a qualitative exploratory design to identify clinician teachers’ needs. Semi-structured, individual interviews were conducted with a total of eight (n=8) module co-ordinators in the third year of the MBChB programme, Stellenbosch University, Faculty of Medicine and Health Sciences.

Results: Results indicated the need for continuous technical and pedagogical support which refers to a longitudinal faculty development approach. Additionally, faculty development should include the support in structuring and rethinking the blended curriculum, as well as assisting in the clinicians’ development in their role and identity as a clinical teacher.

Conclusions: These results reveal the importance of faculty development as a targeted longitudinal approach.

## Introduction

The 21 ^st^ century has led to the emergence of learning technologies, which have increasingly changed teaching and learning practices and how higher education institutions (HEIs) approach teaching and learning ( Chen *et al.,* 2017). Increased pressure is put on HEIs to adapt accordingly ( Bokolo *et al.,* 2020; Steel & Hudson, 2001), requiring teachers to be reflective and re-evaluating their current practices in order to stay up to date to the ever-changing innovations in learning technologies. This requires teachers to identify areas for continuous development.

As part of the emergence of learning technologies, pedagogies such as blended learning have been at the forefront of teaching and learning innovations and have proven to facilitate deep and meaningful learning by actively involving the student in meaningful, interactive activities, i.e. moving towards education that is learning-centred ( García-Cabrero *et al.,* 2018; Garrison & Kanuka, 2004). From the perspective of teaching and learning, blended learning could encourage pedagogical approaches such as active learning strategies, self-directed and adaptive learning, which have proven to engage the student in their learning ( Alebaikan & Troudi, 2010; Collaço, 2017), and improve skills such as critical thinking ( Poon, 2012; Reiss & Steffens, 2010), collaboration ( Castro, 2019) and communication ( Yucel & Usluel, 2016).

Although blended learning has proven to be beneficial in teaching and learning practices, there are challenges in creating and implementing a successful blended learning course which have been well researched ( Dakduk *et al.,* 2018; Smith & Hill, 2019). These challenges include a lack of capacity to develop blended learning courses, resistance to change and paucity of research-informed models to support institutional adoption ( Smith & Hill, 2019).

Faculty development continues to play a key role in enhancing individuals’ teaching ( Steinert, 2020). Literature has been explicit about the need for faculty development in order to implement a blended learning approach ( Dewi *et al.,* 2018; Garrison & Vaughan, 2013). Adopting new instructional and pedagogical strategies, especially after the rapid changing circumstances COVID-19 caused ( Cleland *et al.,* 2020), requires faculty members to participate in faculty development opportunities ( Steinert, 2014; Steinert, 2020; Steinert *et al.,* 2016). Over the past few years, faculty development has increasingly assisted in the development of new trends in teaching and learning and has become a critical part of HPE ( Steinert, 2020). Most educational institutions offer courses and programmes to improve knowledge, skills and behaviour in both individual and group settings ( Steinert, 2014). With the disruption of COVID-19, faculty development offerings were taken online ( Eltayar *et al.,* 2020) in the form of webinars in order to get faculty members up to speed in terms of online teaching and facilitation. Although clinicians engage in teaching and learning practices, many have not undergone formal or informal training, especially in the field of learning technologies. Hence, the role of faculty development has been emphasised in literature in order to assist in implementing a blended learning approach ( Dewi *et al.*, 2018; Garrison & Vaughan, 2013). Faculty development traditionally entails formal programmes which include activities to prepare teachers in terms of knowledge, skills and performance in their respective fields ( Steinert, 2020). However, it seems as if there is a need for more than just a one-off formal workshop and instead more faculty development opportunities that take a longitudinal approach that includes learning through reflection, self-directed learning, and authentic experiences such as observation and workplace learning ( Steinert *et al.*, 2016).

It is important to understand who the clinician teacher is, and what their role and responsibility is when faculty development offerings are proposed. The doctor-teacher role in the context of HPE is indeed interwoven. The typical teacher in the HPE context takes on multiple professional roles, which include being a clinician/practitioner, a teacher and a researcher ( Harden & Lilley, 2018), including additional duties such as keeping up with faculty development and being a role model and facilitator. Many of these clinician teachers’ professional practice and expertise lies outside the education domain and they have little to no educational training, which could make teaching a daunting task ( Swanwick, 2010). Clinician teachers in HPE face the challenge of juggling the theoretical aspect (classroom teaching) and the mastery of critical thinking and clinical reasoning involving the presence of the patients ( Riveros-Perez & Rodriguez-Diaz, 2017). Some clinician teachers identify primarily as doctors, some take a middle ground and others see themselves as teachers ( Higgs *et al.,* 2004). However, the adoption of the role of teacher is essential for quality clinical learning, but the transition from doctor to teacher could be a difficult task ( Riveros-Perez & Rodriguez-Diaz, 2017), as most teachers are not primarily trained to teach ( Swanwick, 2010). Some teachers do attend educational courses, but most do not and have to adjust and develop teaching skills on their own ( Riveros-Perez & Rodriguez-Diaz, 2017).

The aim of this study was to identify and understand the needs of clinician teachers in order to successfully implement a blended learning approach in the health professions curriculum. It is important to note that this study was conducted in 2019 before the World Health Organization (WHO) declared COVID-19 a pandemic and the lockdown period began. COVID-19 had an enormous influence on clinician teachers and their teaching practices.

## Methods

### Study design

In this study, a qualitative research design, based on the interpretative paradigm ( Nieuwenhuis, 2016) was selected. The interpretive paradigm attempts to make sense of multiple worlds, including both ontological and epistemological perspectives ( De Villiers, 2005). Ontologically speaking, interpretivism assumes that multiple truths are considered in a single phenomenon and that it is closely related to one’s view of reality. Interpretivism as an epistemological approach allowed me to view the world of the participants through their perceptions and experiences ( Thanh *et al.,* 2015). This approach gave a comprehensive, holistic understanding of the participants and how they perceive the use of blended learning in their teaching.

### Data collection

The population for this study consisted of the ten module co-ordinators in the third year of the Bachelor of Medicine and Bachelor of Surgery (MBChB) programme. A total population sampling ( Etikan *et al.,* 2016) was used because the ten module chairpersons in this specific population co-ordinate modules which are quite different in nature and this would yield the best representative sample for the study. All the participants were experts in their respective medical fields, with several years of teaching experience both in the clinical and classroom settings. While all the participants, except for one, were medical doctors, they came from a variety of specialties within medicine, which aided in rich data collection. 

The ten module chairpersons were invited via email to take part in the study. The email included the aim, objectives and purpose of the study as well as the informed consent form. The participants who agreed to take part in the study were asked to read through the informed consent form prior to the individual interview, and the form was then signed by both the participant and the researcher on the day of the interview. A total of eight participants agreed to participate in the interviews (n = 8).

Data was collected by means of semi-structured individual interviews, which allowed for an understanding of the participants’ thought processes and gave them the opportunity to explore and reflect on their own experiences ( Holloway & Wheeler, 2010). An interview guide, with some pre-determined questions, assured some kind of commonality, covering the same material in all the interviews ( Brits, 2022). Probing and clarification questions added to the flexibility of the interviews and provided a chance to explore issues that came up spontaneously.

A single interview was conducted per participant which took 30-40 minutes each and most of them were conducted in the Centre for Health Professions Education’s library in the FMHS. Some of the interviews were conducted in participants’ offices in Tygerberg Hospital due to their limited time availability. All the interviews were conducted by the same researcher. The interviews were recorded digitally, transcribed ( Brits, 2022), anonymised and sent to participants for member checking.

### Data analysis

Data analysis and coding were done manually, as the amount of data could be handled without using analysis software. As a point of departure, in order to identify, analyse and report patterns, thematic analysis was used ( Braun & Clarke, 2006). An inductive approach allowed findings in this study to emerge from the raw data ( Strauss & Corbin, 1998) without the restraints of structured frameworks. Codes and themes were identified following Braun and Clarke’s six-step framework ( 2006). This framework provided a structure that allowed for analysis to move beyond describing what participants said to interpreting it.

### Ethical statement

The research was approved by the Health Research Ethics Committee at the Faculty of Medicine and Health Science (FMHS), Stellenbosch University (S19/04/077). The research conformed to the ethical guidelines of the International Declaration of Helsinki. Participation in the study was voluntary and informed written consent was obtained from the participants before data collection commenced. Participants were allowed to end their participation at any time.

## Results

The results indicated that clinician teachers have a number of key needs that seem to influence them to implement a blended learning approach including a need for knowledge of blended learning, a need for confidence in using digital technologies, a need for support and mentoring in the development and implementation of a blended curriculum, and understanding who the clinician as teacher is and what their role and responsibility is.

### Theme 1: A need for knowledge of blended learning

This theme revealed how participants understood the concept blended learning and their experience in using digital technologies in their teaching.

When participants were asked whether they know what blended learning is, it was clear that they had some idea but were uncertain about what blended learning entails and how to implement it in their context.

*“To be honest, I’m a little bit uncertain. I think it means using various technologies to assist learning.” (Lecturer 6)*

It was evident in participants’ responses that not having the practical experience of implementing blended learning, hindered them from attempting to do so.

*“So, in theory, I think I’ve got an idea, but I haven’t experienced much of it, no” (L2).*

Some participants voiced their need for awareness of the possibilities in using digital technologies in teaching and learning. There seems to be a need for exposure to authentic practice-based learning where the teacher experiences the application of blended learning before implementing it themselves.

*“Because I think also you don't know what you don't know. And you don't know the possibilities unless you are exposed to it.”* (L1)

This theme indicated that participants lack knowledge and experience of what blended learning is in a teaching context and that there is a need for exposure to blended learning practices.

### Theme 2: A need for confidence in using digital technologies

A few participants seemed to be quite aware of their lack of confidence and proficiency in using digital technologies which seemed to be a major hindrance to implement a blended learning approach. A need for confidence in one’s own ability to use digital technologies and to feel equipped were identified.

The following participant’s remark reveals the emotions associated with using technology.

*“I feel so incompetent, so I really don't enjoy working with it ... but I know some people have a more natural inclination to work with technology and they enjoy it. ... I don't think I’ve got a natural inclination towards technology, no I suppose I also sort of find it quite, I find it sometimes a little bit embarrassing to admit that I can’t do it.” (L2)*

The frustration of using digital technologies when not fully equipped were evident in some of the participants’ responses. The following quote demonstrates how someone that is competent in their own area of expertise can feel inadequate and frustrated, and how teachers experience stressors differently:

*“I know it sounds so stupid, but we work in like a different world. It’s like you work in this war zone. You see hundreds of patients, and I’m comfortable with that… but then when I sit in front of a computer and I can’t remember my password, or I can’t even get into the module, I get so anxious and so angry.” (L2)*

The fear of failure seemed to discourage some of the participants from implementing learning technologies. This particular participant expressed her experience with using technology as stressful and out of her control.

*“…is nerve-wracking because people can call you on what you don’t know. So, any teaching situation is at baseline a nervous situation, which you can choose to be very prepared for, and try and control every variable. So, you don’t want variables that are out of your control.” (L7)*

In addition to the fear of failure, elements of embarrassment are a factor that can prevent the adoption of blended learning.

*“I need somebody to literally sit and hold my hand I support… I find it sometimes a little bit embarrassing to admit that I can’t do it.” (L2)*

This theme indicates that confidence in using digital technologies plays a vital role in the implementation of a blended learning approach, which could be something that can be addressed by offering faculty development opportunities.

### Theme 3: A need for support and mentoring in the development and implementation of a blended curriculum

Guarding against simply implementing digital technologies in teaching and learning for the sake of it, there is a need to plan a blended learning curriculum. This theme indicates the importance of support and mentoring in the development and implementation phase of a blended learning curriculum.

It is evident from this response that there is a need to learn how to rethink the respective curricula in using a blended learning approach, to have knowledgeable staff to assist in planning the blended curriculum instead of merely rushing into the application thereof.

*“... I need a little bit of guidance in terms of how to start it … have a few practical tips of how to actually start the whole process.”* (L4).

Participants indicated the need for advice on how to approach their blended curriculum planning, re-thinking their teaching and implementation in their specific context. Developing a successful blended learning course requires detailed thinking and planning of the course structure.

*“…I don’t think it even crosses people’s minds to rethink how it’s done, to look at the bigger picture.” (L2)*

Other key needs that were identified in this study is the need for mentoring in the process of planning and implementing a blended learning curriculum. It seems that only attending workshops might not be sufficient.

*“I've been on enough of these lectures and workshops to know a lot of the things that are available... But that doesn't make me comfortable and have insight into what would really work in a specific situation.”* (L7)
*“So I'm obviously reading about stuff and I'm involved and I listen to everything, and I still can’t really picture, sort of on a practical level, exactly what it means to make it blended learning, to be honest with you … I can’t imagine, even if they give me six months just to turn everything into blended learning, I still don't know exactly what I would do.”* (L2)

Therefore, it was clear that participants needed advice, individual assistance and mentoring instead of only one-off faculty development offerings. After-service support, personal attention and relationship building play a critical role in the use of technology and i.e. the implementation of a blended learning approach.

*“I need somebody to literally sit and hold my hand I suppose”* (L2).
*“I really feel actual involvement in the modules by experts from the educational department, people that know about blended learning, will help … In the actual design and planning of the technology, you would actually need enough people who could actually understand your module and sit with you and look through your whole module, and help you to spot places where you can use the technology … a more involved adviser, than just somebody that says please call me when you need anything.”* (L7).

This theme reiterated the importance of planning a blended learning curriculum with the necessary support and mentoring.

### Theme 4: The clinician as teacher

There is a strong reference to clinicians’ dual role as a doctor and teacher. Participants made it clear that they identified as doctors by profession and their role as teacher was in addition to their priorities. In this quote, it is evident that some participants identified as clinicians/doctors and not as teachers.

*“Because us as lecturers, we are primarily, obviously doctors. We’re not teachers. We try to be I'm a doctor, not a teacher [laughs].”* (L7)

Participants had divided responsibilities and commitments, and from the following responses, it was clear that their clinical duties would normally be prioritised.

*“... is the thing of prioritising teaching in the sense of giving it our most important resource as clinicians, is time. So if you want it to be prioritised, you have to give the clinician time to work on it. So if they give me time to work on it, one day a week, one day in two weeks, then you can get something done. But the clinician pressures always push out the rest.” (L2)*
*“... clinical teaching staff get caught up in a spiral, where clinical responsibilities make up the bulk of their time, and teaching is a smaller part of it.”* (L3)

Some participants’ responses indicated that they had not yet fully developed in their teaching practices due to their lack of knowledge about teaching.

*“I think it’s just as I am learning more about teaching, because I never had any educational teaching. It was only the PRONTAC [PREDAC - Professional Educational Development of Academics] that I attended. So it was like you go along and you teach as you go along, what you think is the right thing to teach and how you were taught and so forth.” (L5)*

It is clear from the findings that clinician teachers have certain needs that have to be met in order to implement a blended learning approach. These needs include knowledge of blended learning, confidence in using digital technologies, a need for support in the development and implementation of a blended curriculum, and a need for mentoring in the implementation process. Additionally, the importance of understanding who the clinician as teacher is, was evident.

## Discussion

In this study faculty development opportunities appear to be one of the crucial factors in the implementation of a blended learning approach. Most participants reflected on their lack of knowledge and skills which, according to the literature, is the main challenge in the implementation process ( Dewi *et al.*, 2018; Maarop & Embi, 2016). A lack of knowledge and skills influences teachers' delivery approaches and their willingness to adopt blended learning. However, the implementation of blended learning does not only require one to have technical knowledge, but also pedagogical knowledge and hands-on experience. Garrison & Kanuka (2004) argue that exposing teachers to successful examples of blended learning courses might lead to the successful implementation of a blended learning approach. Studies that support this notion refer to authentic environments that expose the teacher to blended learning experiences ( Herrington *et al.,* 2014; Rowe *et al.,* 2013). Principles of authentic practice-based learning ( Boud, 2012; Yardley *et al.,* 2012) could result in opportunities for learning and professional development if teachers are exposed to relevant experiences. Clinician teachers might not have a sufficient frame of reference when implementing blended learning and might not be able to relate their teaching to good practice when attempting to implement digital technologies in the different learning environments, particularly the clinical learning environment; this influences their acceptance and implementation.

Additionally, a lack of self-efficacy could be due to teachers’ limited digital literacy. Some aspects of Bandura’s social cognitive theory ( 1986) can be drawn on. Self-efficacy can influence the decision to accept change or consider technology use. Bandura (1997) refers to self-efficacy as the belief in one’s own ability to perform a task or behaviour. Some participants lacked the confidence to use new, unfamiliar tools and did not feel equipped to teach with digital technologies. They often referred to not feeling confident in their own ability to use digital technologies in their teaching and learning practices. Similarly to Anderson's findings ( 2012), participants related their lack of confidence (self-efficacy) to implement digital technologies to a lack of knowledge, skills and experience. Anderson (2012) argues that perceived level of self-efficacy plays a role in motivation to use technology. It can be argued that digital literacy could influence confidence in the use of learning technologies. This is in line with work done by Abbitt (2011), who confirms that there is a relationship between teachers' digital literacy skills and self-efficacy and their technology integration. Bandura’s (1986) Social Cognitive Theory (SCT) suggests that increased knowledge will lead to increased self-efficacy, and therefore potentially lead to the increased use of digital technologies in teaching. Based on these findings, the assumption can be made that digital literacy could influence self-efficacy and confidence to implement digital technologies in teaching practices. It is therefore vital that faculty development opportunities address digital literacy.

Participants often referred to attending faculty development opportunities but reported that it was challenging to implement learning technologies in their teaching practices and that they needed hands-on support. This might refer to a need for longitudinal support and mentorship. Steinert *et al.* (2016) describe faculty development as not only being a once-off workshop, but being a longitudinal intervention, including mentoring for one-to-one support. They assert that one of the key features of effective faculty development is relationship building. Similarly to Steinert *et al.* (2016), Balmer & Richards (2012) found that faculty development is more than just reaching the primary goals of gaining knowledge, skills and attitude. Instead, they found that there are secondary goals that need to be addressed such as relationship building. Accumulating different perspectives from colleagues prompts reflection and ultimately allows us to learn from each other’s experiences. Therefore, faculty development should be process-oriented rather than only content-oriented ( Balmer & Richards, 2012) where a focus is placed on relationship building, support, individual attention and active involvement from the institution in blended learning curriculum development ( Sarfo & Yidana, 2016).

The role of the clinician as a teacher and their daily clinical priorities further influence their decision to implement a blended learning approach. Participants identified primarily as being doctors/clinicians and not necessarily as teachers. This assumption is supported by Elmberger *et al.* (2019), who found that their participants did not regard themselves as teachers because they lacked formal training in education. It should be noted that participants in this current study were clinicians by profession with full-time clinical responsibilities. While some participants did not identify as teachers, it could be argued that this could influence their priority to teach. Cantillon, Dornan & De Grave (2019) argue that clinician teachers have to juggle between their professional roles. It was clear that participants in this current study conceptualised their teaching roles in different ways which influenced their teaching motivations and which role they prioritised. It seems that with time constraints, clinician teachers have to prioritise one of their roles over the other, which in general is their clinical roles when, in effect, teaching does not get priority. This aligns with Elmberger *et al.* (2019), who found that activities hold unequal value in their context. Juggling between activities means that time has to be divided between activities, creating tension between hospital duties and university activities ( Cantillon *et al.,* 2016). Participants’ primary identification with their role as doctors/clinicians rather than as teachers could be due to some influences that are found in the literature. Sethi, Ajjawi, McAleer & Schofield (2017) report several tensions and fears that could influence identity formation, such as losing clinician status and the bias of teachers as being undervalued. These fears might assist in understanding why clinician teachers do not identify as teachers. It seems that there is a need to assist clinician teachers in their development and identity as a teacher. The institution has a significant influence on the teachers’ identity ( Lemaire *et al.,* 2017) and it is suggested that faculty development can enhance and motivate clinician teachers to take on a teaching role ( Cantillon *et al.*, 2019).

## Conclusion

Faculty development plays an important role in the implementation of a blended learning approach. It was found that clinician teachers need continuous development, support and mentoring in the implementation process. Development and support are not separate entities and therefore it is recommended that faculty development involve not only one-off workshops, but rather a longitudinal development process with follow-up opportunities for learning, support and mentoring. Teachers need support in the structuring and rethinking of the blended curriculum as well as to be exposed to successful examples of blended learning courses both in the clinical and classroom learning environments. It is therefore important that best practices be shared in the implementation process.

Faculty development seems to support and motivate teachers’ process of development in their role as clinician teacher. The study findings indicate that clinician teachers’ dual role as clinicians and teachers, their multiple responsibilities and their limited time have an influence on their development as teachers. Supportive management needs to focus on professionalisation of the teacher role in an attempt to assist in developing them as teachers, as clinician teachers do not necessarily see themselves as teachers but rather as doctors. Ongoing professional development of clinician teachers is essential where teachers are guided and mentored to implement a blended learning approach.

## Data availability

### Underlying data

Figshare: Implementing blended learning for clinician teachers: Identifying their needs and its impact on faculty development initiatives. https://doi.org/10.6084/m9.figshare.20528154.v4. ( Brits, 2022) 

This project contains the following underlying data:

- Interview transcripts of 8 participants

### Extended data

This project contains the following extended data:

- Interview guide
- Consolidated criteria for reporting qualitative studies (COREQ): 32-item checklist

Data are available under the terms of the Creative Commons Attribution 4.0 International license (CC-BY 4.0).
