# Supplementary material for: Implementing blended learning for clinician teachers: a qualitative study identifying their needs and the impact on faculty development initiatives
Source: MedEdPublish (2016). 2022 Aug 16;12:57. [Version 1] doi: 10.12688/mep.19243.1 (PMC9926507; doi:10.12688/mep.19243.1)
Supplement: Peer review history

# Peer review: Reviewer response for version 1

**DOI:** 10.21956/mep.20616.r32811

**Published:** 2022-12-09

**Authors:** Barbara Jennings

**Affiliations:** 1 Norwich Medical School, University of East Anglia, Norwich, UK

I enjoyed reading this article describing a qualitative study exploring the needs of clinical teachers in their use of technology and blended learning. The professional development needs of clinical teachers alongside the expansion of technology enhanced learning (TEL) in the twenty first century is considered in a thoroughly referenced and engaging introduction.

 The background literature review considered the challenges of the sudden and complex demands (linked to TEL) during the pandemic, and the importance of well-supported longitudinal faculty development that goes beyond one-off formal workshops. Important messages are also presented about the complex transition from a primarily clinical identity to that of the clinical teacher.

 The methodology, research governance, data collection and analysis steps are all clearly described. An additional strength is the commitment to open data sharing and full transparency with respect to methods: the interview guide, COREQ checklist and an extended data set are all made available as files that can be viewed on Figshare: https://doi.org/10.6084/m9.figshare.20528154.v4. An inductive approach is described for the data analysis steps to identify emerging themes – allowing flexibility and opening the possibility of new lines of enquiry in the field.

 The big themes that emerged about what teachers need will resonate with faculty development teams: knowledge of theory and practice of blended learning concepts; confidence in using digital technologies; support and mentoring through implementation; the dual identities for the clinician as teacher.

 The coded quotations were used very effectively to illustrate the main concepts within each theme. I found the exploration of the second theme to be very powerful and felt great empathy for the participants over the many barriers and types of friction that they faced trying to intermittently engage with digital technologies.

 A few minor manuscript problems were noted, e.g., the odd typo should be corrected, e.g., an incomplete sentence in in the fourth paragraph of the introduction; and explicitly say how many researchers completed the data collection and coding in the methods section, and whether any limitations in scope of study or planned data collection or analysis were identified.

 I think this article will be of interest to most clinical teachers; it can serve as supportive evidence that our changing roles are inherently demanding, as well as a reminder of the sorts of professional development activities to seek out. But the research findings and final conclusions will be of particular importance to faculty development leads and programme convenors - and have implications for resource allocation and faculty support.

Have any limitations of the research been acknowledged?

No

Is the work clearly and accurately presented and does it cite the current literature?

Yes

If applicable, is the statistical analysis and its interpretation appropriate?

Not applicable

Are all the source data underlying the results available to ensure full reproducibility?

Yes

Is the study design appropriate and is the work technically sound?

Yes

Are the conclusions drawn adequately supported by the results?

Yes

Are sufficient details of methods and analysis provided to allow replication by others?

Yes

Reviewer Expertise:

Genetic epidemiology; medical education and faculty development.

I confirm that I have read this submission and believe that I have an appropriate level of expertise to confirm that it is of an acceptable scientific standard.

# Peer review: Reviewer response for version 1

**DOI:** 10.21956/mep.20616.r32741

**Published:** 2022-12-09

**Authors:** Anita Samuel

**Affiliations:** 1 Center for Health Professions Education, School of Medicine, Uniformed Services University, Bethesda, MD, USA

This is a qualitative study on clinical educators and their knowledge and implementation of blended learning. The findings indicate a strong need for longitudinal faculty development rooted in a relational approach. The findings of this study can be helpful for those developing faculty development programs and faculty support centers.

 A few small edits to the manuscript can help strengthen it.

- Limitations of this study need to be acknowledged. Even for a qualitative study, 8 is a small sample size. 
- Why were module coordinators selected as participants for this study? Why were the clinician teachers not interviewed directly? An explanation of this is needed. 
- The last question in the interview schedule asks whether the participant would encourage the use of blended learning. This is an interesting question but it has not been addressed in the findings.
- A demographic table for the participants would be helpful. 
- Braun and Clarke use a 6 phase thematic analysis including coding, identifying sub-themes, categorizing into major themes. More information is needed about how the themes were identified. A table might be sufficient for this. 
- In the Study Design section, you say " epistemological approach allowed me to view." Since this is a 3 person author team, the use of 'me' needs to be clarified. 

Have any limitations of the research been acknowledged?

No

Is the work clearly and accurately presented and does it cite the current literature?

Yes

If applicable, is the statistical analysis and its interpretation appropriate?

Not applicable

Are all the source data underlying the results available to ensure full reproducibility?

Yes

Is the study design appropriate and is the work technically sound?

Yes

Are the conclusions drawn adequately supported by the results?

Yes

Are sufficient details of methods and analysis provided to allow replication by others?

Yes

Reviewer Expertise:

Online/distance education, adult education, educational technologies, qualitative research, faculty development.

I confirm that I have read this submission and believe that I have an appropriate level of expertise to confirm that it is of an acceptable scientific standard, however I have significant reservations, as outlined above.

# Peer review: Reviewer response for version 1

**DOI:** 10.21956/mep.20616.r32735

**Published:** 2022-11-28

**Authors:** Simone Titus

**Affiliations:** 1 Interprofessional Education Unit, Faculty of Community and Health Sciences, University of Western Cape, Cape Town, South Africa

Thank you for entrusting me with this review.

 This paper considered the needs of clinical teachers when implementing blended learning approaches as part of professional development in health professions education.

 This study is well worth academic scrutiny in health professions education as the field of HPE and learning with technologies is rapidly evolving. In addition, the current higher education system calls for the need to meet the changing demands of learning and teaching in a bid to enhance academic and student success/performance.

 Blended learning, as the authors indicate, provides an opportunity for students to engage with learning material through both online and face-to-face provision. However, the authors indicate that not all clinical teachers are able to implement blended learning successfully for various reasons.

 The literature used by the authors is relevant as it delves into the context that is relevant and shows that they have reflected on pedagogical approaches to learning, teaching, clinical teaching and blended learning. The literature unpacks the benefits of blended learning and teaching practices and also how the lack of capacity development and support influences the adoption of such practices. Using the literature, the authors aver that faculty development plays a crucial role in enhancing knowledge and skills of blended learning through formal and informal training using educational technologies. The authors offer literature that indicates that the dual, and often multiple roles of clinical teachers are complex within the HPE context, as they are expected to be a clinician, teachers and researchers and as such conducting blended learning may be an additional task for which they require training.

 Thus, in their study, the authors aimed to identify the needs of clinician teachers to implement a blended learning approach in their curriculum. The authors acknowledge that this data was collected before the COVID-19 pandemic and as such the practices of the participants may have shifted since then.

 Using an interpretivist approach, the authors qualitatively collected data from eight clinicians in order to gain insight into their use of blended learning within their learning and teaching practices. The authors offer detailed information with regard to the methods they have followed as well as the clear ethical guidelines. The results of their study indicate that clinicians in their study require knowledge of blended learning and they are uncertain and may not have the requisite experience using it. In addition, they have indicated that their participants suggested they do not have the confidence to use some of the educational technologies available and they require mentoring and support when developing and implementing a blended learning environment within their classroom. Lastly, they found that some of their clinician teachers did not view themselves as teachers, but as clinicians and this may have impacted their lack of implementation of a blended learning approach.

 What is useful in this study is that they are focused on learning-centeredness and not a student or staff-centred approach.

 I am wondering whether the authors have considered any links between the themes as the discussion does not necessarily make the linkages. For instance, is knowledge and confidence linked and may this actually be why they need support and mentoring? If so, this needs to be made explicit in the discussion.

 The results the authors present also indicate a level of anxiety and stress that the use of learning technologies may cause, and this was not addressed in the discussion, and perhaps this is an opportunity missed as these fears and insecurities are very real.

 It would also be useful to offer a profile of the clinician teachers as they are described in the methods section as ‘module chairpersons’ and have ‘several years of teaching experience in both the clinical and classroom setting’. Just like quantitative demographic data tells us about the sample population, a participant profile may do the same in this study. This is also partly because under Theme 2 (confidence), as a way of example, they denote the gender of a participant by using the word ‘her’ [out of her control] and everywhere else the authors write ‘their’. So perhaps if we have a profile, it would mitigate some of these minor nuances.

 The authors use ‘we’ in the abstract, but everywhere else the paper is written more neutrally. Perhaps it would be best to simply state ‘ A qualitative exploratory design was used to identify….’. They may also need to state the limitation of this research as this was not done in the paper.

 The conclusion is well written and may need to consider the implementation of a mentoring programme as well since this was something that emerged from their findings.

 All data has been shared and it is accessible.

 Well done to the authors on a neatly written piece which offers good insight into some of the challenges as well as the fears of clinical teachers.

Have any limitations of the research been acknowledged?

No

Is the work clearly and accurately presented and does it cite the current literature?

Yes

If applicable, is the statistical analysis and its interpretation appropriate?

Not applicable

Are all the source data underlying the results available to ensure full reproducibility?

Yes

Is the study design appropriate and is the work technically sound?

Yes

Are the conclusions drawn adequately supported by the results?

Yes

Are sufficient details of methods and analysis provided to allow replication by others?

Yes

Reviewer Expertise:

I work in the field of Health Professions Education, as well as the use of emerging technologies in higher education.

I confirm that I have read this submission and believe that I have an appropriate level of expertise to confirm that it is of an acceptable scientific standard.
